# Multistoried woodlot based agroforestry system for improved resource utilization and incomes for farmer

**DOI:** 10.1016/j.heliyon.2024.e36096

**Published:** 2024-08-14

**Authors:** M.M. Ali, M.S. Bari, M.T. Rahman, I.J. Sarmin

**Affiliations:** Department of Agroforestry and Environment, Hajee Mohammad Danesh Science and Technology University, Dinajpur-5200, Bangladesh

**Keywords:** Multistoried agroforestry, Sustainable landuse, Economics, Brinjal (Solanum melongena), Potato (Solanum tuberosum)

## Abstract

Diversification of cropping pattern coupled with the development of suitable technology packages is crucial to meet the ever-increasing demand for diversified products and sustained farmers’ incomes. We evaluated different woodlot-based multistoried agroforestry systems for their effectiveness to mitigate the devastating effects of climate change by offering multifaceted benefits. Specifically, the present study aimed to assess the yield and probability of woodlot based multistoried agroforestry system with two vegetables, i.e., potato and brinjal during the period of 2019–2020. The vegetables were planted on the floor of the orchard where pineapple were planted in the same row with the trees. The experiment was laid out in a Randomized Complete Block Design (RCBD) with three replications. The results revealed that the upper-storied woody plants and sole vegetables received 100 % Photosynthetically Active Radiation (PAR) but incident light gradually decreased for brinjal and potato, which were grown at the floor of woody trees. The vegetables experienced 55.85(T_3_), 60.70(T_2_), 66.38(T_1_), and 100 (T_4_) % PAR under different tree crop combinations respectively. In both cases the highest BCR (3.75) and (3.09) was obtained in the ghoraneem + pineapple based multistoried agroforestry system for potato and brinjal production, respectively, which may considered as the best technique for higher production, crop diversification, and maximization of land use efficiency.

## 1 Introduction

The population of Bangladesh is projected to reach 192.6 million by 2050, within a land area of 147,570 square kilometers [1,2]. This rapid population growth exerts tremendous pressure on the country's forestland, with approximately 7300 ha lost annually due to the increasing demand for agricultural land, aquaculture, and homesteads [[3], [4], [5]]. As a result, the forest area covers a mere 13.6 % of the country, significantly below the recommended 25 % required for maintaining ecological balance [6]. These circumstances highlight the urgent need to explore novel approaches for enhancing agricultural productivity and preserving forest resources. Agroforestry, characterized by intercropping between annual crops and permanent trees, has gained attention as a dynamic option for production, especially in response to changing climate conditions [7,[8], [9]]. This system offers numerous socio-ecological advantages, including poverty reduction, employment generation, erosion control, and improved soil health [[10], [11], [12]]. The practice of agroforestry dates back to ancient times in Bangladesh, where farmers planted various trees within their homesteads and agricultural lands. More recently, the adoption of multistoried agroforestry production systems has transformed arable areas into productive landscapes, providing goods for both commercial and domestic use, financial security, and the potential to restore degraded land, thus increasing soil fertility [[13], [14], [15], [16]].

Multistoried agroforestry systems, with optimized resource utilization and enhanced income generation, contribute to sustainable land use and improved livelihoods for farmers [17]. By integrating different tree species, crops, and livestock, these systems create a vertically layered structure that maximizes resource utilization and productivity [18]. The upper layers of trees provide shade, support diverse flora and fauna, and contribute to timber production. Meanwhile, the lower layers can accommodate cash crops, vegetables, or forage for livestock, thereby diversifying income sources and enhancing resource efficiency [19,[20], [21]]. The integration of livestock enables the recycling of organic matter and nutrient cycling, leading to improved soil fertility [22]. In contrast to traditional woodlot management practices, which primarily focus on monoculture timber production, multistoried agroforestry systems maximize resource utilization and improve overall productivity [23]. These systems offer a range of benefits, including enhanced soil fertility [22], improved water retention [24], diversified crop production, increased biodiversity [25], and additional income streams [[26], [27]]. These systems provide a pathway towards sustainable land use and improved livelihoods, while simultaneously addressing the challenges posed by population growth, market fluctuations, and climate variability. However, there is limited research on the impact of multistoried agroforestry on productivity and economic performance of land uses in Bangladesh as a subtropical agrarian country. This study aims to assess the potential of multistoried agroforestry systems in three woodlots, aiming to contribute to the sustainable development of Bangladesh's agricultural sector by increasing production from limited land resources and maximizing agricultural land use.

## 2 Materials and methods

### 2.1 Experimental design

**Fig. 1:**
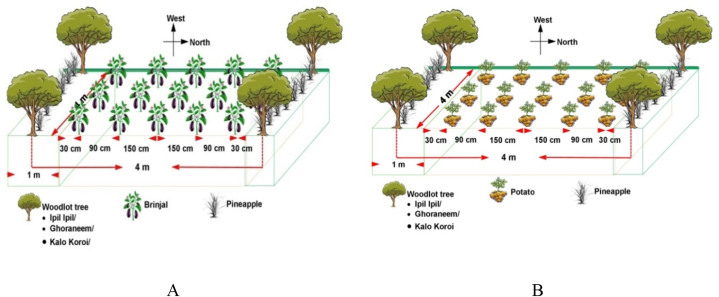
Details layout of the experimental plot with brinjal (A) and potato (B).

The experiment was conducted at the Agroforestry Farm of Hajee Mohammad Danesh Science and Technology University, Dinajpur, during the 2019–2020 season. This farm is located between 25° 13′ latitude and 88° 23′ longitude and about 37.5 m above sea level. The experimental plot was on medium-high land belonging to the Old Himalayan Piedmont Plain area and the soil texture was sandy loam with soil pH of 5.1. The research was conducted in the three 7-year-old woodlot of Ghoraneem (*Melia azedarach*), Kalokoroi (*Albizia lebbeck*), and Ipilipil (*Leucaenia leucocephala*) trees, spaced 4 m × 4 m apart that occupied the upper layer. These excellent multipurpose and deciduous species were chosen for their diverse benefits. As the middle- or second-layer plant, pineapples (var. Honey queen) were planted along the tree rows (Fig. 1) that were one year old and in fruit bearing condition. Brinjal and potato vegetables, considered lower-story crops, were grown in the alleys between the trees. The experiment was laid out in a single factor Randomized Complete Block Design (RCBD) with three replications. The plot size for vegetable cultivation of each treatment was 3 m width x 6 m long with adjacent plots separated by respective tree lines and 0.5 m space remaining free from the tree base. All the tree woodlots had an adjacent open field to its west where brinjal, potato and pine apple was grown as sole crop maintaining same plot size. The sole stand of ipil-ipil, goraneem and kalokoroi were present to the north of multistoried field. The treatments were applied as shown below (Table 1 and Fig. 1).

**Table 1:** Details of the experimental treatments.

System 1 (Brinjal as lower-story crops)	System 2 (Potato as lower-story crops)
T_1_ = Ipilipil + Pineapple + Brinjal	T_1=_ Ipilipil + Pineapple + Potato
T_2_ = Goraneem + Pineapple + Brinjal	T_2=_ Goraneem + Pineapple + Potato
T_3_ = Kala Koroi + Pineapple + Brinjal	T_3=_ Kalokoroi + Pineapple + Potato
T_4_ = Brinjal sole cropping	T_4_ = Potato sole cropping

Schematic layout of the Experimental plot.

### 2.2 Data collection

Harvesting of brinjal and potato was done from 15 February to 22 March and 24 February. Data in respect of various parameters such as plant height, number of leaves, primary branch per plant, fruit per plant, fruit weight, tuber weight, etc., were recorded as per the vegetable's yield. Ten plants were collected randomly from each plot. Yield per plot was converted to t/ha. Plants in the outer rows and at the end of the middle rows were excluded from the random selection to avoid a border effect. Photosynthetically active radiation (PAR) was measured on each vegetable row using LP-80 Accu PAR Ceptometer to determine the extent of shading by the tree species. Such measurement was done at 9.30 a.m., 12.30 p.m., and 3.30 p.m. each day at a one-week interval.

**Benefit-cost ratio (BCR) and** Land equivalent ratio (**LER) calculation:**

Benefit-cost ratio (BCR) and land equivalent ratio (LER) were determined according to the equations described present the equation by Alam (2018) in a woodlot-based multistoried agroforestry system.

Benefit-cost ratio (BCR) = Gross return/Total cost of production.

Land equivalent ratio (LER) = Ci/Cs + Ti/Ts.

where,

Ci is crop yield under agroforestry,

Cs is crop yield under sole cropping,

Ti is fruit yield under agroforestry, and.

Ts is fruit yield under sole cropping.

### 2.3 Statistical analysis

The data on statistically analyzed to determine the significant variations of the results due to different multilayered agroforestry systems. The analysis of variance for each of the studied characters was done by the F (variance ratio). These data were analyzed statistically following the ANOVA and means were adjudged by LSD test at 1 % and 5 % levels of significance and a regression analysis was carried out to get an insight into the PAR-induced influence on crop yields. All data were processed, calculated, and analyzed using computer software MS Excel and STATISTIX 10.

## 3 Results and discussion

### 3.1 Availability of photosynthetically active radiation (PAR) in different multistoried agroforestry systems

The highest photosynthetically active radiation (PAR) was observed in open field conditions (T_4_), with 980.15 μmol m^−2^ S^−1^ at 9:30 a.m., 1245.92 μmol m^−2^ S^−1^ at 12:30 p.m., and 874.71 μmol m^−2^ S^−1^at 3:30. Light interception by the tree canopy and competition for light pose limitations to the success of component crops in any agroforestry systems. This is evident in Table 2, which presents light incidence in different combinations of ipil-ipil, goraneem, kalokoroi, pineapple, brinjal, and potato. The lowest PAR occurred in the kalokoroi, pineapple, and potato/brinjal combination (T_3_), with 451.05 μmol m^−2^ S^−1^at 9:30 a.m., 898.76 μmol m^−2^ S^−1^ at 12:30 p.m., and 381.03 μmol m^−2^ S^−1^at 3:30 p.m. Consequently, the highest average daily PAR (1033.61 μmol m^−2^ S^−1^) was also observed in open field conditions. The upper-storied woody plants received 100 % PAR, but the light incidence gradually decreased for brinjal and potato in multistoried arrangements. Vegetables in open fields received 100 % PAR. As canopy coverage increased, light intensity decreased. The overstory canopy's varying sizes and shapes led to different light levels in different systems [28]. Among the three trees, the ipil-ipil has the lightest canopy due to its smaller leaves, allowing more light to penetrate to the ground. In contrast, the kalakoroi has a denser canopy, which hinders light penetration.

**Table 2:** Photosynthetically Active Radiation (PAR) in different combinations of multistoried agroforestry systems.

Treatments	PAR Average			Daily Average light (μmol m^−2^ S^−1^)	% PAR compared to open field
	9:30 a.m.	12:30 a.m.	3:30 a.m.		
T_1_ = Ipilipil + pineapple + potato/brinjal	665.31b	893.57b	498.28b	658.72b	66.38 %
T_2_ = Goraneem + pineapple + potato/brinjal	556.01c	875.13b	450.2c	627.11b	60.70 %
T_3_ = Kala Koroi + pineapple + potato/brinjal	451.05d	898.76b	381.03d	576.94c	55.85 %
T_4=_ Brinjal and potato open field	980.15a	1245.92a	874.71a	1033.61a	100 %
LSD _(0.01)_	25.93	44.15	16.30	33.67	–
CV (%)	1.96	2.26	1.48	2.23	–

### 3.2 Performance of brinjal under different multistoried agroforestry systems

The impact of different tree-crop combinations on the yield-contributing characters of brinjal is shown in Table 3. The greatest number of fruits per plant (23.78) was seen in an open field setting and gradually declined as the light levels decreased. This was observed in treatment T_2_ (21.77). Treatment T_3_ had the lowest number of fruits (16.46), which was significantly lower compared to other treatments. Under open field conditions, brinjal produced the largest fruits (15.28 cm) compared to other production systems. Fruits of medium size (14.06 cm) were produced under treatment T_2_, which was significantly smaller than those produced in the open field. The shortest fruits (11.96 cm) were recorded under treatment T_3_, which was significantly shorter than other production systems. As the light levels decreased, the individual fruit weight was also declined. The maximum fruit weight (73.50 g) was found under the open field production system, followed closely by treatment T_2_ (71.28 g). Treatment T_3_ had the lowest fruit weight (56.38g), which was significantly lower. Similarly, the dry fruit weight gradually declined with the decrease in light levels. The maximum dry weight (5.52g) was observed under open field conditions and was similar to treatment T_2_ (5.06g). The minimum dry weight (3.82g) was recorded under treatment T_3_, which was significantly lower than the rest of the treatments. The highest yield per plant (1.792 kg) was seen under open field conditions and was like treatment T_2_ (1.588 kg). The lowest yield (0.953 kg) was recorded under treatment T_3_, which was significantly lower than other treatments. A similar trend in brinjal yield was noted by Miah (2000) [29] This situation manifests as stunted growth, fewer branches, and smaller leaves, all of which contribute to lower fruit production. Furthermore, light plays a significant role in the flowering and fruit set processes in brinjal. Reduced light intensity disrupts these processes, resulting in fewer flowers, poor pollination, and ultimately, fewer fruits. Hossain et al. [18] showed a significant decrease in brinjal yield with increasing canopy size and decreasing light intensity no yield was obtained when brinjal was cultivated under 12-year-old litchi trees, indicating the critical role of light in brinjal production. Kabir [30] investigated the effect of different light levels on the growth and yield of brinjal and the findings revealed a clear negative correlation between light intensity and yield. Brinjal varieties grown under full sunlight (100 % Photosynthetically Active Radiation - PAR) produced the highest yield, while yields decreased significantly under reduced light conditions.

**Table 3:** Yield and yield contributing characters of brinjal under multistoried agroforestry systems.

Treatments	BRINJAL					
	No. fruit/plant	Length of fruit (cm)	Fresh wt. of fruit (g)	Dry wt. of fruit (g)	Yield/plant (kg)	Yield (ton/ha)
T_1_ = Ipilipil + Pineapple + Brinjal	18.90	13.24	67.48	4.74	1.389	17.83
T_2_ = Goraneem + Pineapple + Brinjal	21.77	14.06	71.28	5.06	1.588	20.61
T_3_ = Kalo koroi + Pineappl + Brinjal	16.46	11.96	56.38	3.82	0.953	10.71
T_4=_ Brinjal as sole cropping	23.78	15.28	73.50	5.52	1.792	23.51
LSD _(0.01)_	3.84	0.98	6.08	0.47	0.294	2.89
CV (%)	15.88	10.05	11.32	15.02	25.04	14.86

### 3.3 Performance of potato under different multistoried agroforestry system

The highest yield (29.20 ton/ha) of potato was seen in the Ghoraneem + Pineapple + Potato (T_2_) based agroforestry system, which was statistically the same as the sole cropping (28.42 ton/ha) of potatoes (Table 4). On the other hand, the lowest yield (18.76 ton/ha) was recorded in the Kalakoroi + Pineapple + Potato (T_3_) based agroforestry system. The production of potatoes increased by 2.75 % under the Ghoraneem + Pineapple + Potato (T_2_) based agroforestry system over control the yield of potatoes decreased by 34 % under the Kalakoroi + Pineapple + Potato (T_3_) based agroforestry system compared to sole cropping of potatoes. The highest yield was due to the maximum rate of photosynthesis under Ghoraneem trees, which may have been due to the deciduous nature of these trees, allowing light to penetrate the canopy and provide adequate light for maximum photosynthesis. Additionally, the decomposition of tree litter added organic matter to the soil, ensuring maximum production. This result agrees with the findings of Malik et al. [31] who noted a higher yield of potatoes under eucalyptus trees. Singh [32] also reported that the potato yield was 22 tons/ha under a guava-based agroforestry system.

**Table 4:** Growth and final tuber yield of potatoes under different tree-crop patterns.

Treatments	POTATO			
	Fresh wt. of haulm per hill (g)	Number tuber per hill	Weight of tuber of per hill (g)	Yield (ton/ha)
T_1_ = Ipilipil + Pineapple + Potato	66.87	8.37	257.7	18.68
T_2_ = Goraneem + Pineapple + Potato	70.80	10.10	353.3	29.20
T_3_ = Kalo koroi + Pineapple + Potato	80.60	6.67	144.3	18.76
T_4=_ Potato as sole cropping	57.33	8.00	403.3	28.42
LSD _(0.01)_	7.45	1.62	58.98	4.21
CV (%)	4.27	11.71	17.06	8.97

### 3.4 Performance of pineapple as sole and second story crop

The cultivation of pineapples as a sole crop and as a second-story crop, offering insights into several key parameters. In the sole crop scenario, pineapples have more leaves (27.2) compared to the second-story crop (23.5), indicating potentially better vegetative growth in the former. However, pine apple as the second-story crop exhibits slightly smaller leaf size (278 cm^2^) compared to the sole crop (311 cm^2^). The fruit characteristics in the second-story crop are notably different, with smaller fruit dimensions (11.30 cm in length and 9.15 cm in breadth and 872.60 g in weight) compared to the sole crop (13.88 cm, 11.60 cm, and 1.07 kg, respectively). Because of these differences, the yield for the second-story crop (27.9 tons/ha) was lower than that of the sole crop (41.45 tons/ha), which is around 33 % less compare to the sole crop (Table-5). These results suggest that the second-story approach led to smaller individual fruits, and had a lower overall yield compared to the traditional sole crop method. Rana et al.,[33] also found this type of result in an experiment of mango-pineapple based agroforestry systems in madhupur, Bangladesh.

**Table 5:** Performance of pineapple as sole and second story crop in multistoried agroforestry.

Cultivation process	No. of leaves	Leaf size (cm^2^)	Fruit length (cm)	Fruit breath (cm)	Fruit weight(kg)	Yield (ton/ha)
Sole	27.20	311.00	13.88	11.60	1.07	41.45
Second story crop	23.50	278.00	11.30	9.15	0.87	27.90
LSD _(0.01)_	3.50	5.50	0.90	1.40	0.11	3.50
Cv (%)	6.97	13.12	9.51	4.98	13.26	9.67

### 3.5 Relationship between light intensity (% PAR) of agroforestry system and yield of brinjal and potato

**Fig. 2:**
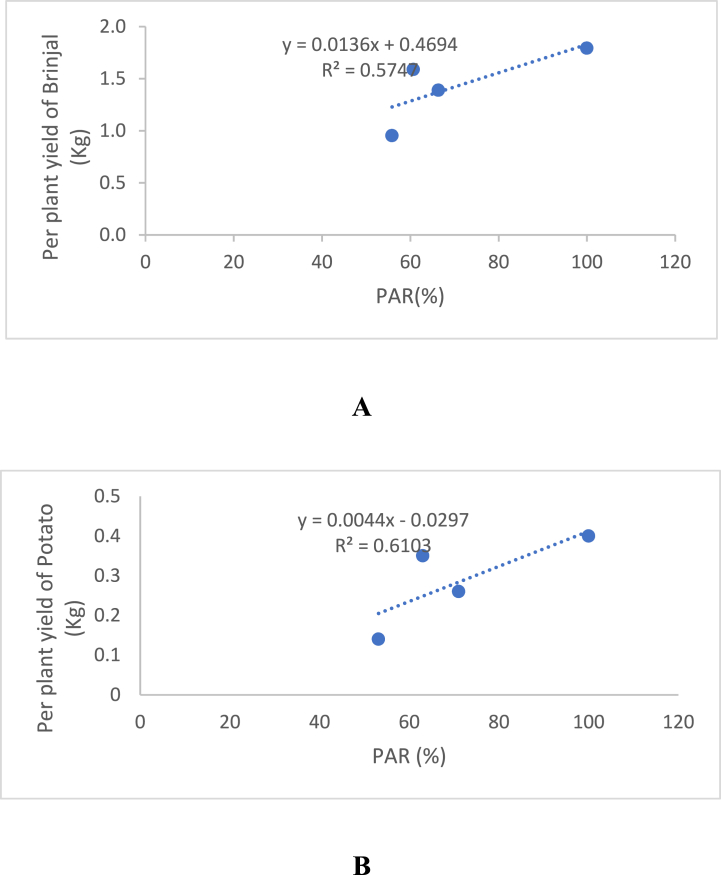
Relationship between PAR (%) and yield of Brinjal (A) and Potato (B) in woodlot-based multistoried Agroforestry systems.

The correlations between photosynthetically active radiation (PAR) and the yield of brinjal and potato in different agroforestry systems based on woodlots was analyzed and illustrated in Fig. 2 (A and B). A positive linear relationship was found between PAR and the yield of both crops. The relationship between brinjal yield and PAR was represented by equation Y = 0.0136x + 0.4694, with a high and significant R^2^ value of 0.5747, indicating that PAR could explain 57.47 % of the variation in brinjal yield. The equation also showed that the maximum yield of brinjal occurred at 60.70 % PAR and that beyond this level, the yield decreased by 0.0136 kg per plant for every unit change in PAR. These findings are consistent with the findings of Kabir et al. [34]. The relationship between PAR and potato yield and expressed as Y = 0.0044x - 0.0297, with a high and significant R^2^ value of 0.6103, suggesting that PAR accounted for 61.03 % of the variation in potato yield. The equation also indicated that the maximum yield of potato was achieved at 63.01 % PAR, and beyond this level, the yield decreased by 0.0044 kg per plant for every unit change in PAR. Schulz et al. [35] also found that availability of PAR increase potato yield.

### 3.6 Benefit-cost ratio of agroforestry production

The highest benefit-cost ratio of 3.63 was recorded form the Ghoraneem + Pineapple + Potato (T_2_) system, followed by the Ipil-Ipil + Pineapple + Potato (T_1_) based system and the Kala koroi + Pineapple + Potato (T_3_) system. The lowest benefit-cost ratio of 2.14 was observed in the sole cropping of potato (T_4_). For brinjals, Ghoraneem + Pineapple + Potato (T_2_) was the most profitable treatment with a BCR of 3.07, driven by a high gross return of 577114 Tk./ha (Table-6). These findings are similar to those of [36] who reported benefit-cost ratios ranging from 2.35 to 3.73 in a poplar + potato-based agroforestry system.

**Table 6:** Economics of potato and brinjal production under the different tree based agroforestry system.

Treatments		Return (Tk./ha)					Gross Return (Tk./ha)	Total cost of Production (Tk./ha)	Net Return (Tk./ha)	BCR
		Potato /Brinjal	Ipil-Ipil	Ghora neem	Kala koroi	Pine apple				
Potato field	T1	140100	83438	–	–	218700	442238	138939	303299	3.18
	T2	219000	–	70312	–	214530	503842	138687	365155	3.63
	T3	140700	–	–	93750	211870	446320	139758	306562	3.19
	T4	284200	–	–	–	–	284200	132651	151549	2.14
Brinjal field	T1	267450	83438	–	–	209812	560700	186399	374301	3.01
	T2	309150	–	70312	–	197652	577114	188062	389052	3.07
	T3	160650	–	–	93750	185230	439630	191476	248154	2.30
	T4	470200	–	–	–	–	470200	183870	286330	2.56

N.B. Potato 10 Tk./kg, Brinjal 20 Tk./kg, Pineapple 30 tk/kg Ipil-Ipil 267 Tk./Tree/Year, Ghoraneem 225 Tk./Tree/Year, Kala koroi 300 Tk./Tree/Year. For Potato T_1_ = Ipilipil + Pineapple + Potato, T_2_ = Goraneem + Pineapple + Potato, T_3_ = Kalo koroi + Pineapple + Potato and T_4=_ Potato as sole cropping. For Brinjal T_1_ = Ipilipil + Pineapple + Brinjal, T_2_ = Goraneem + Pineapple + Brinjal, T_3_ = Kalo koroi + Pineappl + Brinjal and T_4=_ Brinjal as sole cropping. The vegetable production season was considered for six months. Therefore, return from trees and pineapple was divided by two. Moreover, for vegetable production under multistoried agroforestry systems as ground layer crops, 75 % land of 1 ha was considered in return calculation.

## 4 Conclusion

In this study, it is evident that light availability, plays a crucial role in shaping crop performance. The research reveals that open field conditions consistently provide the highest PAR levels, leading to superior crop yields. As the canopy coverage and the complexity of the agroforestry systems increase, PAR decreases, impacting annual crop growth. Brinjal and potato, two key crops under investigation, exhibited notable reductions in yield and fruit size as PAR levels diminished. But these systems offer higher returns due to diversified products compared to sole cropping. The Ghoraneem + Pineapple + Potato and Ghoraneem + Pineapple + Brinjal combinations emerged as particularly promising, demonstrating the potential for optimizing land use and income generation within these systems. Further research is needed to explore the long-term sustainability and socioeconomic effects of multistoried agroforestry systems in Bangladesh in order to develop a vertical agriculture policy tosupport sustainable agricultural development.

## CRediT authorship contribution statement

**M.M. Ali:** Writing – original draft, Visualization, Validation, Supervision, Software, Resources, Methodology, Investigation, Formal analysis, Data curation, Conceptualization. **M.S. Bari:** Writing – review & editing, Supervision, Methodology, Investigation, Conceptualization. **M.T. Rahman:** Writing – original draft, Visualization, Software, Methodology, Formal analysis, Data curation. **I.J. Sarmin:** Writing – original draft, Visualization, Validation, Software, Resources, Methodology, Formal analysis, Data curation.

## Declaration of competing interest

The authors declare no conflict of interest in conducting and presenting the above-mentioned research article titled "Multistoried Woodlot Based Agroforestry System for Better Resource Utilization and More Income Generation for Farmer." There are no financial, personal, or professional relationships with organizations or individuals that could bias or be perceived to influence the research findings and interpretations. The study is undertaken with scientific integrity, and the authors have no competing interests that may impact the objectivity or impartiality of the reported results. This declaration is made to ensure transparency and uphold ethical standards in the presentation of the research outcomes.

## References

[1] BBS (2018). Ministry of Planning, Govt. Of Peoples Republic of Bangladesh.

[2] United Nations, Department of Economic and Social Affairs, Population Division (2019). World population prospects and probabilistic population projections based on the world population prospects. http://population.un.org/wpp/.

[3] Khan S. (2019). The financial express online desk. https://www.thedailystar.net/backpage/un-report-says-242m-underfed-bangladesh-1772470.

[4] Muhsin N., Ahamed T., Noguchi R. (2018). GIS-based multi-criteria analysis modeling used to locate suitable sites for industries in suburban areas in Bangladesh to ensure the sustainability of agricultural lands. Asia Pac. J. Reg. Sci..

[5] Rahman M.M., Haque M.A., Nihad S.A.I., Howlader N.M. H. Akandand M.R. A. (2016). Morpho-physiological response of *Acacia auriculiformis*as influenced by seawater induced salinity stress. Off. Syst..

[6] BBS (2009).

[7] Campanhola C., Pandey S., Campanhola C., Pandey S. (2019). Sustainable Food and Agriculture.

[8] Ali M.M., Ahmad B., Bari M.S., Pal A.C., Rahman M.L., Sarmin I.J. (2024). An assessment of agroforestry as a climate‐smart practice: evidences from farmers of northwestern region of Bangladesh. Agrosystems, Geosciences & Environment.

[9] Djanibekov U., Dzhakypbekova K., Chamberlain J., Weyerhaeuser H., Zomer R., Villamor G., Xu J. (2016).

[10] Singh S.K., Sharma M., Singh P.K. (2016). Combined approach of intercropping and INM to improve availability of soil and leaf nutrients in fruit trees. J. Chem. Pharm. Sci..

[11] Udawatta R.P., Gantzer C.J., Jose S., Al-Kaisi M.M., Lowery B. (2017). Soil Health and Intensification of Agroecosystems.

[12] Miah M.G., Islam M.M., Rahman M.A., Ahamed T., Islam M.R., Jose S. (2018). Transformation of jackfruit (*Artocarpus heterophyllus* Lam.) orchard into multistory agroforestry increases system productivity. Agrofor. Syst..

[13] Adane F., Legesse A., Weldeamanuel T. (2019). The contribution of a fruit-tree-based agroforestry for household income to smallholder farmers in Dale district, Sidama zone, Southern Ethiopia. Adv. Plants. Agric. Res..

[14] Gomes C., Garcia E., Alves E., Queiroz M., Oliveira andF.P., Gupta R.K., Ajay-Gulati P.K. (2015).

[15] Qiao X., Sai L., Chen X., Xue L., Lei L. (2019). Impact of fruit-tree shade intensity on the growth, yield, and quality of intercropped wheat. PLoS One.

[16] Rahman M.S., Roy P.R., Ali M.M., Bari M.S., Sarmin I.J., Rahman M.A. (2020). Cost-benefit analysis of different agroforestry systems and practices of Kaharole Upazila of Dinajpur District, Bangladesh. South Asian Journal of Social Studies and Economics.

[17] Ali M.M., Islam M.A., Islam M.R., Dipto S.S., Bari M.S. (2024). Assessing the cropland changes into agroforestry and its livelihood outcomes: evidence from northern Bangladesh. Trees, Forests and People.

[18] Hossain S., Rahman M.S., Kona K.N., Bari M.S., Akter N., Ali M.M. (2019). Growth performance of two ginger (zingiber officinale roscoe) varieties under different agroforestry systems in Bangladesh. Asian Plant Research Journal.

[19] Ali M.M., Rahman M.M., Islam S., Islam M.A., Alam M.R., Bari M.S., Nahar M.N. (2018). Varietal performance of turmeric under mango based agroforestry system. Am. J. Plant Sci..

[20] Bari M.S., Roshetko J.M., Ali M.M., Hasan M.F. (2024). Potential of agroforestry practices in multifunctional landscapes for enhancing the livelihoods of local dwellers in the north-western charlands of Bangladesh. Forest and Society.

[21] Abedin M.Z., Quddus M.A. (1990).

[22] Kumar, C., C. Saint-Laurent, S. Begeladze and M.Calmon(ed.) Enhancing Food Security through Forest Landscape Restoration: Lessons from Burkina Faso, Brazil, Guatemala, Viet Nam, Ghana, Ethiopia and Philippines. IUCN: Gland, Switzerland. 42–69.

[23] Sileshi G.W., Dagar J.C., Nath A.J., Kuntashula E., Dagar J.C., Gupta S.R., Sileshi G.W. (2023). Agroforestry for Sustainable Intensification of Agriculture in Asia and Africa. Sustainability Sciences in Asia and Africa.

[24] Nair P.R., Kumar B.M., Nair V.D., Nair P.R., Kumar B.M., Nair V.D. (2021). Other ecosystem services of agroforestry. An introduction to agroforestry: four decades of scientific developments.

[25] Van Noordwijk, Dagar J.C., Gupta S.R., Teketay D. (2020). Agroforestry for Degraded Landscapes.

[26] Das A.K., Rahman M., Keya S.S., Saha S.R. (2020). Malta-based agroforestry system: an emerging option for improving productivity, profitability and land use efficiency. Environmental Sustainability.

[27] Jose S., Udawatta R.P., Udawatta R.P., Jose S. (2021). Agroforestry and Ecosystem Services.

[28] Ferdous J. (2021).

[29] Miah M.M. (2000). Performance of five winter vegetables under different light conditions for agroforestry systems. MS thesis.

[30] Kabir M. (2020). Effect of different levels of light intensity on morphophysiology and yield of brinjal (Solanum melongena L.) [Master's thesis, Sher-e-Bangla Agricultural University]. SAU Institutional Repository.

[31] Malik M.S., Surendran C., Kailasham K. (2005). Predicting growth of *Eucalyptus* globulus under agroforestry plantation. IndianJ.For..

[32] Singh R.A. (2007). Productivity and employment generation through guava based agroforestry system in gangetic area of. U.P. Range-Management-and-Agroforestry.

[33] Rana M. (2022). Productivity analysis of mango based agroforestry systems in the madhupur sal forest of Bangladesh. European Journal of Agriculture and Food Sciences.

[34] Kabir M., Hossain M.F., Hasanuzzaman M., Habib Z.F.B., Mahmud J.A. (2020). Effect of different levels of light intensity on morphophysiology and yield of brinjal (Solanum melongena L.). J. Sher-e-Bangla Agric. Univ..

[35] Schulz V.S., Munz S., Stolzenburg K., Hartung J., Weisenburger S., Graeff-Hönninger S. (2019). Impact of different shading levels on growth, yield and quality of potato (Solanum tuberosum L.). Agronomy.

[36] Mbow C., Van Noordwijk M., Luedeling E., Neufeldt H., Minang P.A., Kowero G. (2014). Agroforestry solutions to address food security and climate change challenges in Africa. Curr. Opin. Environ. Sustain..

